# Associations between waist circumference and executive function among Chinese Tibetan adolescents living at high altitude

**DOI:** 10.3389/fnut.2023.996785

**Published:** 2023-03-20

**Authors:** Yuan Liu, Feng Zhang, Leimin Gan, Lijuan Shi, Xiaojian Yin, Yaru Guo, Pengwei Sun

**Affiliations:** ^1^Key Laboratory of Adolescent Health Assessment and Exercise Intervention of the Ministry of Education, East China Normal University, Shanghai, China; ^2^College of Physical Education and Health, East China Normal University, Shanghai, China; ^3^Department of Physical Education, East China University of Political Science and Law, Shanghai, China; ^4^Research Department of Primary Education and Preschool Education, Shanghai Teacher Training Center, Shanghai, China; ^5^College of Economics and Management, Shanghai Institute of Technology, Shanghai, China

**Keywords:** waist circumference, executive function, Tibetan, high altitude, adolescents

## Abstract

**Background:**

Associations between body composition and execution function (EF) were currently studied in low altitude (LA) areas. However, the research on the correlation between waist circumference (WC) and EF among adolescents living at high altitude (HA) was limited.

**Objective:**

We sought to explore the association between WC and EF in Chinese Tibetan adolescents aged 13–18 years in HA areas.

**Methods:**

After excluding invalid data and extreme values, 1,228 participants (583 boys and 645 girls) were eventually included. The areas of Lhasa (average elevation of 3650 m), Nagqu (4500 m), Qamdo (3500 m), and Nyingchi (3100 m) in China were chosen as study sites. Participants completed tasks to measure inhibitory control, working memory, and cognitive flexibility. The predictive association between WC and EF was explored by One-way ANOVA, Pearson correlation, and linear regression analysis.

**Results:**

After controlling for concomitant variables, the reaction time (RT) of responding to inhibitory control (difference incongruent and congruent), working memory (1-back, 2-back), and cognitive flexibility (heterogeneous, difference in heterogeneous and homogeneous) stimuli in subjects with WC ≥ 85th percentile was longer than that in those with WC of the 15th percentile or below [by 1.785 ms (95% CI: 0.078, 3.491), 208.734 ms (95% CI: 96.886, 320.582), 106.679 ms (95% CI: 16.485, 196.873), 82.307 ms (95% CI: 19.171, 145.442), and 58.397 ms (95% CI: 0.343,116.452), respectively], (*P* < 0.05).

**Conclusion:**

After adjustment for concomitant variables, WC was significantly positively associated with the RT of inhibitory control, working memory, and cognitive flexibility among Chinese Tibetan adolescents in HA areas.

## 1. Introduction

Executive function (EF) is an umbrella term for the related but distinct processes involved in the effortful control of goal-directed behavior (1), including three main factors, namely inhibitory control, working memory, and cognitive flexibility (2). Its purpose is to regulate and control the cognitive process (3). EF is known to be particularly sensitive to oxygen; studies have shown that the EF of people who live in high altitude (HA) areas with low temperatures and hypoxia may be impaired (4–6). Recently, the health question of humans in HA areas has become a topic under increasing discussion, and the impact of HA exposure on cognition has attracted the attention of scholars. Long-term exposure to HA areas may lead to cognitive deficits, such as in inhibitory control, working memory, and cognitive flexibility (7–9). Zhu et al. showed that the cognitive level of students in HA areas was relatively lower than that of students in low altitude (LA) areas, postulated to be mainly driven by chronic hypoxia in HA areas (10). Long-term exposure to HA areas has been shown to negatively affect individuals’ spatial working memory and significantly reduce their inhibitory control (11, 12).

As one of the factors influencing EF and underlying brain developmental processes in adolescents, body composition may be statistically correlated with EF (13–15). Soaring obesity rates and declining physical health have become among the most serious public health concerns worldwide (16, 17), notably in places as diverse as the United States (18, 19), Serbia (20), and China (21). Meanwhile, recent evidence suggests that obesity may have adverse health effects. Obesity has also been associated with cognitive performance across the life cycle, particularly with negative associations with EF (22, 23). As an effective indicator of abdominal obesity mass, waist circumference (WC) is used to define abdominal obesity (24), reflecting the accumulation of fat in the abdomen and valid predictor of future cardio-metabolic and chronic diseases (25). Even after controlling for body mass index (BMI), high WC appeared to be particularly detrimental to metabolic regulation, affecting type 2 diabetes risk (26). Most studies have focused on the relationship between BMI and EF (27, 28), but, there have been few studies on the correlations between WC and EF in HA areas.

At present, the correlation between WC and EF was not very uniform. Some studies have confirmed that high WC was negatively associated with EF (29, 30). In structural equation modeling in adolescents, WC through a higher metabolic risk factor cluster score (MetS-cluster score) and through lower high-density lipid cholesterol (HDLc) displayed a statistically significant negative relationship with reaction time (RT) of incongruent stimuli (29). Research has also found that the combined adverse effects of high WC on academic performance were observed in both boys and girls for grade point average (GPA) indicator (30). However, some other studies found that high WC was positively correlated EF (31). The results were inconsistent with other research regarding the relationship between WC and EF, which found that higher WC predicted higher cognitive ability among Indian children aged 9–10 years (31). The relationship between WC and EF remains unclear.

Most previous studies have focused on the relationship between BMI and EF. However, the research on the relationship between WC and EF remains limited. In addition, previous studies have mainly focused on adolescents in LA areas (32), while there have been few studies on the relationship between WC and EF among adolescents in HA areas. As one of the highest-altitude populations in the world, making it more likely to observe differences among Chinese Tibetan adolescents in HA areas. Hence, this paper sought to clarify the independent and joint correlations among WC and EF of Tibetan adolescents living in HA areas.

## 2. Materials and methods

### 2.1. Data sources and participants recruitment

**FIGURE 1:**
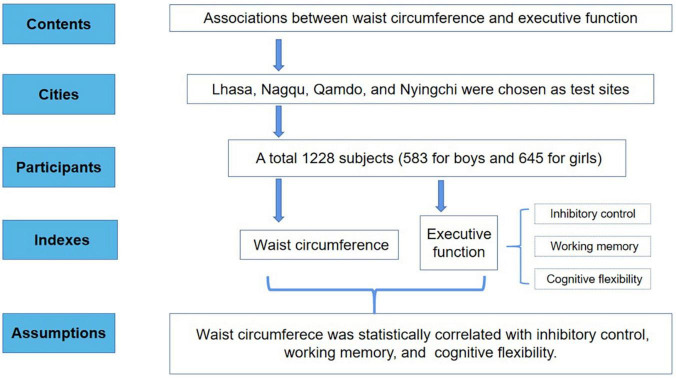
Flowchart of the study design.

A stratified random cluster sampling method was adopted to identify test sites determined by surveys on the physical health of Tibetan students. Lhasa, Nagqu, Qamdo, and Nyingchi were chosen as test sites for this study (with average altitudes of 3650, 4500, 3500, and 3100 m, respectively). After performing the EF test, the valid data were the ones whose accuracy rate of each task reached over 80%. The specific inclusion criteria of the participants were people aged 13–18 years without color blindness or serious physical or mental illness. After excluding invalid data and extreme values, based on a gender ratio of approximately 1:1, data from 1,228 subjects (583 for boys and 645 for girls) were collected for this analysis in the Tibet Autonomous Region in China from August 2019 to December 2020 (Figure 1).

### 2.2. Waist circumference

Waist circumference was measured according to the specific requirements of the physical health survey of students in China (33). In the test, the participants were asked to stand upright and breathe gently. The investigators faced the participants and put the nylon tape 1 centimeter (cm) above the navel on the horizontal plane of the waist. The line of sight was on the same level as the nylon tape, and the reading was in cm (accuracy: two decimal place). The test error could not exceed 0.01 cm.

### 2.3. Body mass index

Body mass was measured to the nearest 0.1 kg on an electronic scale (Tanita BWB-800S, Tanita Corporation, Tokyo, Japan). Stature was measured to the nearest 0.5 cm using a portable stadiometer (SECA 214, Seca Corporation, Hamburg, Germany). BMI was calculated according to the results of the recorded height and weight test using the standard formula. BMI = weight (kg)/height (m^2^) (33).

### 2.4. Executive function and related assessments

The EF task-cueing paradigm was that developed by Aiguo et al. (34), including inhibitory control test developed by Flanker (35), working memory test (36), and cognitive flexibility test (37). The RT for a correct test was recorded as the final result, and a shorter RT reflected a better performance. The RTs of the congruent and incongruent exercise were used to show inhibitory control. The 1-back and 2-back tests were used to indicate working memory. The difference in RTs between heterogeneous and homogeneous blocks was used to estimate cognitive flexibility. The EF tests were conducted on a computer using a program created by the E-prime 1.1 software system (Psychology Software Tools Inc., Pittsburgh, PA, USA).

#### 2.4.1. Inhibitory control

The Flanker task involved two types of tests, congruent and incongruent. Participants gaze at the center of the computer screen for 500 ms, and then a series of five capital letters appear on the screen for 1000 ms. One of two different situations appear randomly: one situation is congruent (LLLLL or FFFFF) and the other incongruent (LLFLL or FFLFF). The center of the screen alternates with a + sign, and each stimulus appears at an interval of 2 s. Participants press either the “F” or “L” key with their left or right index finger. The test was divided into pre-test practice, stage 1, and stage 2. The pre-test practice was 12 rounds of the judgment response task, and stage 1 and stage 2 included 48 rounds each. The inhibitory control was calculated as the difference in response time between incongruent and congruent stimuli.

#### 2.4.2. Working memory

The 1-back task displayed five uppercase letters (A, S, P, G, T) in the center of the screen. The participants attempted to accurately remember the letters that appeared by pressing the “F” key if the letter appeared consistent with the previous letter, or the “J” key if it was inconsistent. The formal test was divided into two stages of 25 repetitions each. The stimulus interval for each capital letter was 3 s, and the time for the letter to appear on the screen was 2000 ms. In this study, the test age of 1-back was 10–18 years.

The task if the 2-back exercise was to indicate whether the letter in the center of the screen was consistent with the one letter in front, pressing the “F” key quickly if it was, or pressing the “J” key if not. Other test requirements and methods were the same as with the 1-back test.

#### 2.4.3. Cognitive flexibility

This experimental test was divided into three parts. Part 1: At the beginning of the task, a black Arabic number (1–4 and 6–9, in random order) continuously appeared in the center of the computer screen. Participants judged the size of a “5” by pressing the D key if the digit was smaller than the 5 and pressing the F key if the digit was larger than the 5. The final result for each participant was their average RT across all trials. Part 2: A green number (1–4 and 6–9, in random order) appeared in the center of the screen at the beginning of the task. Participants pressed the J key if the digit was odd and pressed the K key if it was even. The final result for each participant was his average RT across all tasks. The heterogeneous conditions comprised Parts 1 and 2. Part 3 was then conducted: This test randomly interspersed the tasks used in Part 1 and Part 2. That is, if the number was black, the participant judged the size of the 5 (by pressing the D key if it was smaller and the F key if it was larger, in both cases using the left hand). If the number was green, participants judged whether it was an odd or even number (by pressing the J key if it was odd and the K key if it was even, in both cases using the right hand). A participant’s final result was their average RT across all trials (i.e., homogeneous RT). The whole test consisted of six stages, which were conducted in the order of A, B, C, C, B, A. A was Part 1 (i.e., the size judgment), and was performed 16 times in total. B was the parity judgment, Part 2, and was performed 16 times in total. C was the size/parity judgment, Part 3, which was performed 32 times in total. A practice round was not included in the test score; it was performed eight times before Part 1 and Part 2, and 16 times before Part 3.

### 2.5. Statistical analyses

**FIGURE 2:**
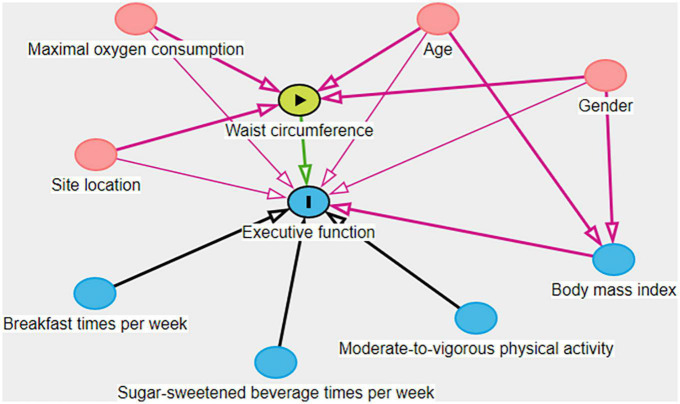
Directed acyclic graph of the associations between waist circumference and executive function.

Waist circumference was categorized according to percentile by the Lambda Mu Sigma (LMS): very low (WC < 15th percentile), normal (15th percentile ≤ WC < 85th percentile), high (WC ≥ 85th percentile) (38). Many studies have demonstrated that EF could be affected by obesity rate (22, 23), dietary intake (39), physical activity (40), and maximum oxygen uptake (VO_2max_) (41). Therefore, sociodemographic and dietary intake information, BMI, WC, moderate-to-vigorous physical activity (MVPA), and VO_2max_ were used as covariates in this study. Based on previous studies and data in this study on the relationship between WC and EF in adolescents, directed acyclic graph (DAG) was used to identify possible covariates on exposure, and outcomes (Figure 2) (42). Three models were developed for this study: Model 1 was conducted after adjusting for demographic indicators (gender, age, BMI, and site location) by questionnaires. Based on Model 1, Model 2 controlled for MVPA, VO_2max_. Physical activity status was obtained by the following two questions: “In the past 7 days, how many times did you have MVPA on school days and weekends, respectively?” If students answered more than 0 times, they were further asked about the duration each time, “On average, how long does each activity last?” The VO_2max_ was estimated by 20 m SRT and the details measurement of 20 m SRT was provided in our previous study (38). Based upon Model 2, Model 3 included breakfast, sugar-sweetened beverage intake as additional variables. The associations between WC and EF were evaluated using one-way ANOVA, *Pearson* correlation, and linear regression analysis. Data were analyzed using IBM SPSS 25.0 software (IBM Corp., Armonk, NY, USA); the test significance level was α = 0.05. Results were considered statistically significant if *P* < 0.05.

## 3. Results

### 3.1. Descriptive characteristics for various variables

Among the 1,228 Tibetan adolescents studied, there were 583 boys (47.48%) and 645 girls (52.52%), with an average age of 15.77 ± 1.678 years. The average WC of children and adolescents aged 13–18 years was 68.21 ± 7.13 cm. Grouped by WC, the proportions of subjects in the <15th percentile WC group, the ≥ 15th and <85th percentile WC group, and the ≥ 85th percentile WC group were 16.69, 70.85, and 12.46, respectively. The average values of congruent, incongruent, 1-back, 2-back, heterogeneous, and homogeneous were 774.94, 794.51, 965.67, 1078.05, 744.15, and 1125.69 ms, respectively (Table 1).

**TABLE 1:** Characteristics and assessments of adolescent subjects.

Variable	Total sample	Standard error	IQR	Kurtosis	Skewness	Coefficient variable
*N*	1228					
Boys (%)	583 (47.48)					
Girls (%)	645 (52.52)					
Age	15.77 ± 1.68	0.048	3.00	-1.166	-0.215	10.64%
Waist circumference (cm)	68.21 ± 7.13	0.201	7.40	3.629	1.231	10.32%
WC < percentile 15 (%)	205 (16.69)					
WC ≥ percentile 15 and < percentile 85 (%)	870 (70.85)					
WC ≥ percentile 85 (%)	153 (12.46)					
BMI (kg/m^2^)	20.38 ± 2.68	0.076	3.15	1.835	0.919	13.15%
Underweight (%)	63 (5.13)					
Normal weight (%)	1020 (83.06)					
Overweight (%)	115 (9.36)					
Obesity (%)	30 (2.45)					
VO_2max_ (mL/kg/min)	38.37 ± 7.82	0.232	12.01	-0.599	0.011	20.37%
Moderate-to-vigorous physical activity per week (h)	4.42 ± 0.85					
Breakfast times per week (times)	2.89 ± 0.40					
Sugar-sweetened beverage times per week (times)	1.41 ± 0.59					
**Inhibitory control (ms)**						
Congruent (ms)	774.94 ± 84.43	2.409	107.07	0.063	0.576	10.90%
Incongruent (ms)	794.51 ± 83.76	2.390	104.28	0.078	0.595	10.54%
Difference in incongruent and congruent (ms)	19.55 ± 6.52	0.186	11.03	-1.268	0.116	33.36%
**Working memory (ms)**						
1-back (ms)	965.67 ± 429.13	12.246	490.59	246.192	10.803	44.44%
2-back (ms)	1078.05 ± 377.96	10.786	621.04	-0.94	-0.092	35.06%
**Cognitive flexibility (ms)**						
Homogeneous (ms)	744.15 ± 123.04	3.511	198.67	-0.798	0.244	16.54%
Heterogeneous (ms)	1125.69 ± 266.37	7.601	307.08	-0.307	0.707	23.66%
Difference in heterogeneous and homogeneous (ms)	381.55 ± 231.02	6.592	272.66	0.099	0.437	60.55%

WC, waist circumference; BMI, body mass index; VO_2max_, maximum oxygen uptake.

### 3.2. Performance of different WC percentile groups in EF-measuring tasks

All measures of EF index [including inhibitory control RT, working memory RT (1-back, 2-back), and cognitive flexibility RT] differed significantly by WC percentile groups (*F* = 5.479, 11.165, 8.63, and 9.315, respectively, *P* < 0.05). The RTs of EF (inhibitory control, working memory, and cognitive flexibility) increased with WC percentile in different groups (Table 2).

**TABLE 2:** Status of executive function by WC percentile groups.

RT (ms)	Waist circumference	*N*	Mean	SD	Partial η^2^	Cohen’s^f^	*F*	*P*
**Inhibitory control**								
Congruent	WC < percentile 15	153	763.85	78.34	0.006	0.075	3.402	0.034^a^
	WC ł percentile 15 and < percentile 85	870	774.1	82.05				
	WC ł percentile 85	205	786.81	96.92				
Incongruent	WC < percentile 15	153	782.42	78.35	0.007	0.083	4.199	0.015^a^
	WC ł percentile 15 and < percentile 85	870	793.53	81.27				
	WC ł percentile 85	205	807.65	95.88				
Difference in incongruent and congruent	WC < percentile 15	153	18.57	6.11	0.009	0.095	5.479	0.004^b^
	WC ł percentile 15 and < percentile 85	870	19.44	6.55				
	WC ł percentile 85	205	20.77	6.54				
**Working memory**								
1-back	WC < percentile 15	153	845.12	266.8	0.018	0.135	11.165	0.000^b^
	WC ł percentile 15 and < percentile 85	870	964.67	470.09				
	WC ł percentile 85	205	1059.91	305.65				
2-back	WC < percentile 15	153	972.55	368.13	0.014	0.119	8.63	0.000^b^
	WC ł percentile 15 and < percentile 85	870	1082.7	376.3				
	WC ł percentile 85	205	1137.05	378.32				
**Cognitive flexibility**								
Homogeneous	WC < percentile 15	153	727.69	120.54	0.006	0.076	3.51	0.030^b^
	WC ł percentile 15 and < percentile 85	870	742.9	123.79				
	WC ł percentile 85	205	761.69	120.14				
Heterogeneous	WC < percentile 15	153	1079.95	239.39	0.019	0.141	12.171	0.000^b^
	WC ł percentile 15 and < percentile 85	870	1115.13	266.12				
	WC ł percentile 85	205	1204.68	272.15				
Difference in heterogeneous and homogeneous	WC < percentile 15	153	352.26	220.33	0.015	0.123	9.315	0.000^b^
	WC ł percentile 15 and < percentile 85	870	372.22	233.67				
	WC ł percentile 85	205	442.99	217.76				

RT, reaction time.

^a^*P* < 0.05.

^b^*P* < 0.01.

### 3.3. Bivariate correlations between study variables

Table 3 showed the analysis of the double variable pearson correlations of the various variables of the subjects. There were no mean differences by gender in performance of executive function variables (*P* > 0.05). In general, the higher the WC, the longer the congruent RT, incongruent RT, difference in incongruent and congruent RT, 1-back RT, 2-back RT, homogeneous RT, heterogeneous RT, and difference in heterogeneous and homogeneous RT (*r* = 0.095, 0.102, 0.085, 0.092, 0.122, 0.076, 0.148, and 0.130, respectively, *P* < 0.05). In other words, there was a positive correlation between WC and inhibitory control, working memory, and cognitive flexibility RT, with shorter RT indicating better EF.

**TABLE 3:** Bivariate correlations between variables among Tibetan adolescents in China.

	1	2	3	4	5	6	7	8	9	10	11	12	13
Age (1)	1												
Gender (2)	0.031	1											
Site (3)	0.308^b^	0.070^a^	1										
BMI (4)	0.250^b^	0.239^b^	0.095^b^	1									
WC (5)	−0.018	−0.107^b^	−0.130^b^	0.590^b^	1								
VO_2max_ (6)	−0.211^b^	−0.448^b^	0.071^a^	−0.185^b^	−0.175^b^	1							
RT congruent (7)	−0.224^b^	0.036	−0.196^b^	−0.054	0.095^b^	−0.398^b^	1						
RT incongruent (8)	−0.237^b^	0.033	−0.203^b^	−0.052	0.102^b^	−0.398^b^	0.997^b^	1					
RT difference in incongruent and congruent (9)	−0.144^b^	−0.039	−0.071^a^	0.03	0.085^b^	0.041	−0.143^b^	−0.066^a^	1				
RT 1-back (10)	−0.124^b^	−0.031	−0.04	0.011	0.092^b^	−0.113^b^	0.206^b^	0.205^b^	−0.025	1			
RT 2-back (11)	−0.207^b^	−0.009	−0.166^b^	0.005	0.122^b^	−0.208^b^	0.249^b^	0.255^b^	0.05	0.273^b^	1		
RT homogeneous (12)	−0.125^b^	−0.007	−0.132^b^	−0.011	0.076^b^	−0.270^b^	0.393^b^	0.396^b^	−0.01	0.256^b^	0.304^b^	1	
RT heterogeneous (13)	−0.072^a^	−0.007	−0.088^b^	0.034	0.148^b^	−0.381^b^	0.404^b^	0.406^b^	−0.012	0.175^b^	0.290^b^	0.499^b^	1
RT difference in heterogeneous and homogeneous(14)	−0.017	−0.004	−0.032	0.045	0.130^b^	−0.296^b^	0.256^b^	0.258^b^	−0.008	0.066^a^	0.172^b^	0.043	0.887^b^

BMI, body mass index; WC, waist circumference; VO_2max_, maximum oxygen uptake; RT, reaction time.

^a^*P* < 0.05.

^b^*P* < 0.01.

### 3.4. The multiple linear regression of executive function with different WC percentile groups

The correlation between WC and executive function is presented in Table 4. After controlling for concomitant variables, the RTs of inhibitory control (difference incongruent and congruent), working memory (1-back, 2-back), and cognitive flexibility (heterogeneous, difference in heterogeneous and homogeneous) stimuli in subjects with WC ≥ 85th percentile was longer than that in those with WC of the 15th percentile or below [by 1.785 ms (95% CI: 0.078, 3.491), 208.734 ms (95% CI: 96.886, 320.582), 106.679 ms (95% CI: 16.485, 196.873), 82.307 ms (95% CI: 19.171, 145.442), and 58.397 ms (95% CI: 0.343,116.452), respectively], (*P* < 0.05). In the RTs of inhibitory control evalution, the subjects in the WC ≥ 85th percentile rate significantly different in the inhibitory control compared to those in the WC ≥ 15th percentile and <85th percentile (*P* < 0.05).

**TABLE 4:** The multiple linear regression of executive function by WC percentile groups (*n* = 1228).

RT (ms)	WC	Model 1	Model 2	Model 3
**Inhibitory control**				
Congruent	WC < percentile 15	0 (Reference)	0 (Reference)	0 (Reference)
	WC ł percentile 15 and < percentile 85	14.098 (-0.433 28.629)	12.58 (-0.645 25.806)	13.088 (-0.156 26.331)
	WC ł percentile 85	31.854 (11.609 52.098)^b^	9.354 (-9.642 28.350)	9.467 (-9.525 28.460)
Incongruent	WC < percentile 15	0 (Reference)	0 (Reference)	0 (Reference)
	WC ł percentile 15 and < percentile 85	14.755 (0.401 29.110)^a^	13.434 (0.408 26.460)^a^	13.99 (0.949 27.030)^a^
	WC ł percentile 85	33.678 (13.679 53.678)^b^	11.14 (-7.570 29.849)	11.276 (-7.426 29.977)
Difference incongruent and congruent	WC < percentile 15	0 (Reference)	0 (Reference)	0 (Reference)
	WC ł percentile 15 and < percentile 85	0.654 (-0.496 1.804)	0.855 (-0.333 2.042)	0.904 (-0.286 2.094)
	WC ł percentile 85	1.745 (0.143 3.348)^a^	1.762 (0.056 3.467)^a^	1.785 (0.078 3.491)^a^
**Working memory**				
1-back	WC < percentile 15	0 (Reference)	0 (Reference)	0 (Reference)
	WC ł percentile 15 and < percentile 85	128.362 (52.815 203.908)^b^	123.963 (46.143 201.784)^b^	123.938 (45.947 201.929)^b^
	WC ł percentile 85	247.089 (141.834 352.343)^b^	209.68 (97.906 321.454)^b^	208.734 (96.886 320.582)^b^
2-back	WC < percentile 15	0 (Reference)	0 (Reference)	0 (Reference)
	WC ł percentile 15 and < percentile 85	105.681 (40.250 171.113)^b^	100.836 (38.022 163.650)^b^	100.895 (38.003 163.786)^b^
	WC ł percentile 85	151.559 (60.398 242.720)^b^	107.829 (17.608 198.049)^a^	106.679 (16.485 196.873)^a^
**Cognitive flexibility**				
Homogeneous	WC < percentile 15	0 (Reference)	0 (Reference)	0 (Reference)
	WC ł percentile 15 and < percentile 85	16.525 (-5.191 38.242)	16.517 (-4.676 37.709)	18.155 (-3.034 39.344)
	WC ł percentile 85	36.954 (6.698 67.210)^a^	23.146 (-7.293 53.585)	23.909 (-6.478 54.297)
Heterogeneous	WC < percentile 15	0 (Reference)	0 (Reference)	0 (Reference)
	WC ł percentile 15 and < percentile 85	39.242 (-7.810 86.295)	32.48 (-11.461 76.420)	34.468 (-9.556 78.493)
	WC ł percentile 85	135.369 (69.813 200.925)^b^	81.264 (18.152 144.377)^a^	82.307 (19.171 145.442)^a^
Difference in heterogeneous and homogeneous	WC < percentile 15	0 (Reference)	0 (Reference)	0 (Reference)
	WC ł percentile 15 and < percentile 85	22.717 (-18.362 63.796)	15.963 (-24.405 56.332)	16.313 (-24.168 56.794)
	WC ł percentile 85	98.415 (41.182 155.648)^b^	58.119 (0.137 116.100)^a^	58.397 (0.343 116.452)^a^

RT, reaction time; WC, waist circumference; Model 1, adjusting for gender, age, BMI, and site location; Model 2, adjusting for moderate-to-vigorous physical activity per week, maximum oxygen uptake; Model 3, adjusting for breakfast times per week, sugar-sweetened beverage times per week.

^a^*P* < 0.05.

^b^*P* < 0.01.

## 4. Discussion

This study showed that WC was negatively associated with the inhibitory control, working memory, and cognitive flexibility among Chinese Tibetan adolescents in HA aeras. The RT of inhibitory control (difference incongruent and congruent), working memory (1-back, 2-back), and cognitive flexibility (heterogeneous, difference in heterogeneous and homogeneous) stimuli in subjects with WC ≥ 85th percentile was longer than that in those with WC of the 15th percentile or below, respectively. There were no mean differences by sex in performance EF variables, consistent with findings in prior research (43, 44).

In terms of inhibitory control, after controlling for concomitant variables, WC was negatively correlated with inhibitory control among Tibetan adolescents in HA areas in China. The associations between WC and inhibitory control in the study were generally consistent with those of previous studies. A study found that WC was negatively correlated with inhibitory control (*P* = 0.008) in Danish adolescents (23). At the same time, in the structural equation model, WC was indirectly positively correlated with incongruent RT through higher metabolic risk factor cluster (MetS-cluster) score and lower HDLc. The only statistically significant direct relationship between WC and inhibitory control was for the incongruent RT in the model including HDLc as a mediator (29). Longitudinal associations between inhibitory control and body composition throughout childhood found that better inhibitory control predicted lower subsequent obesity at each measured time point (45). Recent prospective studies (46) reported evidence suggesting that low inhibitory control in adolescents increases the risk of gaining more weight and WC. Interestingly, this paper found that high WC values the factors that predisposes the development of low inhibitory control.

In this study, after adjustment in concomitant variables, the RT of working memory (1-back, 2-back) in the highest percentiles of WC was slower than among those in the lowest percentiles of WC. WC was negatively correlated with working memory of children and adolescents. There have been numerous studies on the associations between WC and working memory that were consistent with the results of this study. Khan et al. explored the associations between abdominal adipose and hippocampal memory forms among pre-pubertal children (7–9-year-olds, *n* = 126) and found that total abdominal adipose tissue had a significant negative association with hippocampal-dependent relational memory behavioral accuracy (47). Hassevoort et al. found that central adiposity was negatively correlated with hippocampal-dependent relational memory among 7–10-year-old children (*n* = 40) who completed a task designed to assess relevant memory performance (*P* < 0.05) (48). After adjusting for multiple factors, we observed lower visuospatial function, executive performance, and language scores in the abdominal obesity group (WC ≥ 90 cm for men and ≥85 cm for women) compared with those in the non-abdominal obesity group, which was supported by the negative correlation between WC and visuospatial function (49). Some studies have linked obesity to an increased risk of memory problems in children (50). Gonzales found that larger WC was associated with decreased associated with diminished working-memory-related blood oxygen level-dependent (BOLD) response in the right superior frontal gyrus (β = −0.008, *P* = 0.001, 95% CI: −0.012 to −0.004) (51).

However, the results were not in accord with a previous southern Indian study of the relationship between WC and working memory performance (31). This cross-sectional study of 540 children reported that after adjusting for age, sex, and socioeconomic factors, higher WC predicted greater cognitive ability (i.e., long-term retrieval/storage, memory, reasoning, verbal abilities, attention, and concentration) in South India (31). The reasons for the discrepancy between this study and previous results may be that the subjects lived in Tibetan HA areas and were therefore exposed to chronic hypoxia (4–6). A study found that acute short-term exposure to HA areas could cause significant WM deficits in healthy children; this effect was particularly significant and more severe in children who lived at HA for a long time (7). The response accuracy of the HA group was significantly reduced in the linguistic and spatial working memory tasks compared with that of the LA group (4). Furthermore, obese children did not demonstrate significant retrieval-induced forgetting (RIF), whereas RIF was present in children (8–12-year-olds) without obesity and in adolescents (13–18-year-olds) with and without obesity (44). One longitudinal study showed that better working memory in grade three predicted lower subsequent body composition in grade four (45).

After adjustment for concomitant variables, WC was negatively associated with cognitive flexibility (heterogeneous, difference in heterogeneous and homogeneous) among subjects living at HA in this study. To support this theory, cognitive flexibility was impaired in obese adolescents (52, 53). Cross-sectional and longitudinal studies involving performance-based cognitive tasks have shown that overweight children and adolescents exhibit poorer cognitive flexibility than do their healthy-weight peers. These findings demonstrated that selective alterations in specific components of cognitive flexibility of overweight adolescents (52, 53). The relationship between WC and cognitive flexibility remains to be confirmed by longitudinal studies. Currently, there is a lack of relevant longitudinal studies on this topic, but Tomaso et al. conducted a longitudinal study on the relationship between cognitive flexibility and body composition (45). A longitudinal study showed that better SF in third grade predicted lower subsequent BMI in fourth grade, but, BMI did not predict subsequent cognitive flexibility performance at any point in time (45).

The results in this study were basically consistent with those of previous studies that also reported a negative correlation between high WC and EF of adolescents. A relatively consistent finding was that obesity was associated with poor EF across the life cycle, particularly in children and adolescents (54). One investigation found that obese (high visceral adipose tissue, H-VAT) children performed significantly lower on cognitive function tests than did normal-weight children (13). Another found that obesity (in terms of waist-hip ratio) in children and adolescents was negatively correlated with all areas of cognitive control (27). However, these results were reversed in the southern Indian study of the relationship between WC and cognitive performance noted above (31). Other studies have differed as well. One study found that Raven colored progressive matrices (CPM) scores were poorer among students with either the lowest or highest body fat percentile (BF%) than among other BF% groups (*P* < 0.05) (55).

Therefore, there is no consensus about the correlation between WC and EF. Improvement in EF levels has been associated with weight loss among overweight and obese adolescents (56), suggesting that EF could be improved and have a positive impact on weight loss (57). EF may have a protective effect on weight problems, especially in young adolescents when these abilities have had more time to develop and children begin to gain more independence (45). The findings suggested that the adverse association between obesity and EF may be attributed to visceral fat (VF), rather than fat stored elsewhere in the body (28). Both obesity and cognitive function could have strong associations with environmental influencing factors (socioeconomic status), though those associations can vary considerably in different directions depending on the setting (58). The results of multiple studies highlight the necessity of developing comprehensive adolescent health plans that promote both healthy body composition and brain function in the future.

### 4.1. Strengths and limitations

Many previous studies have compared WC and executive function in adolescents; nevertheless, their datasets have generally been small and unrepresentative. The strengths of this study include its large sample size (*N* = 1228) for WC and use of multiple-aspect executive function tasks among adolescents living at HA in China. Most previous studies have focused on the associations between BMI and EF, and without regard to elevation; few systematic studies have examined the relationship between WC and EF in HA areas.

This study has some limitations to note. First, this study did not include participants at LA, and we did not compare the differences between the LA and HA. Second, this was a cross-sectional investigation, and therefore could not discern a causal relationship between WC and EF. Third, the investigation of influencing factors in this study was largely based on the participants’ recall ability and attitude, which inevitably led to certain deviations in the survey results. In the future, objective instruments could be used for accurate testing of physical activity parameters.

## 5. Conclusion

In general, after adjustment for concomitant variables, WC was significantly positively associated with the RT of inhibitory control, working memory, and cognitive flexibility tasks measuring executive function among Chinese Tibetan adolescents in HA areas. The reasons for these results should be further investigated by longitudinal studies. Furthermore, effective measures should be taken to reduce obesity and improve EF among adolescents in the HA areas of Chinese Tibet. Further investigation is needed to provide an evidence base for efforts to improve the physical health and EF of adolescents in these areas.

## Data availability statement

The original contributions presented in this study are included in the article/supplementary material, further inquiries can be directed to the corresponding author.

## Ethics statement

Written informed consent was obtained from the minor(s)’ legal guardian/next of kin for the publication of any potentially identifiable images or data included in this article.

## Author contributions

YL and XY: conceptualization. YL and LG: methodology. XY: validation, supervision, and funding acquisition. YL: formal analysis, visualization, resources, writing—original draft preparation, and project administration. YL and LS: investigation. YG and PS: data curation. YL and FZ: writing—review and editing. All authors read and agreed to the published version of the manuscript.

## References

[1] Allan J McMinn D Daly MA. Bidirectional relationship between executive function and health behavior: evidence, implications, and future directions. *Front Neurosci.* (2016) 23:386. 10.3389/fnins.2016.00386 27601977 PMC4993812

[2] Funahashi S. Neuronal mechanisms of executive control by the prefrontal cortex. *Neurosci Res.* (2001) 39:147–65. 10.1016/s0168-0102(00)00224-8 11223461

[3] Chen A Yin H Jun Y Yu Y. Effects of acute aerobic exercise of different intensity on executive function. *Acta Psy Sin.* (2011) 43:1055–62.

[4] Ma H Zhang D Li X Ma H Wang N Wang Y. Long-term exposure to high altitude attenuates verbal and spatial working memory: evidence from an event-related potential study. *Brain Behav.* (2019) 9:1256. 10.1002/brb3.1256 30891949 PMC6456776

[5] Yan X Zhang J Gong Q Weng X. Adaptive influence of long term high altitude residence on spatial working memory: an fMRI study. *Brain Cogn.* (2011) 77:53–9. 10.1016/j.bandc.2011.06.002 21767899

[6] Virues-ortega J Bucks R Kirkham FJ Baldeweg T Baya-Botti A Hogan A. Bolivian children living at altitude project (BoCLA 06). Changing patterns of neuropsychological functioning in children living at high altitude above and below 4000 m: a report from the Bolivian children living at altitude (BoCLA) study. *Dev Sci.* (2011) 14:1185–93. 10.1111/j.1467-7687.2011.01064.x 21884333

[7] Rimoldi S Rexhaj E Duplain H Urben S Billieux J Allemann Y Acute and chronic altitude-induced cognitive dysfunction in children and adolescents. *J Pediatr.* (2016) 169:238–43. 10.1016/j.jpeds.2015.10.009 26541425

[8] Virués-Ortega J Garrido E Javierre C Kloezeman K. Human behaviour and development under high-altitude conditions. *Dev Sci.* (2006) 9:400–10. 10.1111/j.1467-7687.2006.00505.x 16764613

[9] Yan X. Cognitive impairments at high altitudes and adaptation. *High Alt Med Biol.* (2014) 15:141–5. 10.1089/ham.2014.1009 24949527

[10] Zhu X Chen Z Qi H. Relationship between cognitive level and oxygenated hemoglobin content of high school students in high altitude area of China. *Chn High Altitude Med Biol.* (2020) 41:165–71.

[11] Ma H-L Mo T Zeng T-A Wang Y. Long-term exposure to high altitude affects spatial working memory of migrants: evidence from temporal and frequency-domain analyses. *Acta Phy Sin.* (2020) 72:181–9. 10.13294/j.aps.2020.0012 32328612

[12] Ma H Ting M Yan W. Effects of long-term high altitude exposure on spatial working memory of Tibetan residents: evidence from time-frequency analysis. *Chn High Altitude Med Biol.* (2020) 41:88–93. 10.13452/j.cnki.jqmc.2020.02.003

[13] Raine L Drollette E Kao S Westfall D Chaddock-Heyman L Kramer A The associations between adiposity, cognitive function, and achievement in children. *Med Sci Sports Exerc.* (2018) 50:1868–74. 10.1249/MSS.0000000000001650 29727406 PMC6095815

[14] Laurent J Watts R Adise S Allgaier N Chaarani B Garavan H Associations among body mass index. Cortical thickness, and executive function in children. *JAMA Pediatr.* (2020) 174:170–7. 10.1001/jamapediatrics.2019.47 31816020 PMC6902097

[15] Li H Chen A Li W Zhao Q Wang J. Effect of body mass index on executive function in elementary school students. *Chin J Sch Health.* (2016) 37:1834–6. 10.16835/j.cnki.1000-9817.2016.12.023

[16] Spinelli A Buoncristiano M Kovacs V Yngve A Spiroski I Obreja G Prevalence of severe obesity among primary school children in 21 european countries. *Obes Facts.* (2019) 12:244–58. 10.1159/000500436 31030201 PMC6547273

[17] GBD 2015 Obesity Collaborators Afshin A Forouzanfar M Reitsma M Sur P Estep K. Health effects of overweight and obesity in 195 countries over 25 years. *N Engl J Med.* (2017) 377:13–27. 10.1056/NEJMoa1614362 28604169 PMC5477817

[18] Wang Y Beydoun M Min J Xue H Kaminsky L Cheskin L. Has the prevalence of overweight, obesity and central obesity levelled off in the United States? Trends, patterns, disparities, and future projections for the obesity epidemic. *Int J Epidemiol.* (2020) 49:810–23. 10.1093/ije/dyz273 32016289 PMC7394965

[19] Hales C Carroll M Fryar C Ogden C. Prevalence of obesity among adults and youth: United States, 2015-2016. *NCHS Data Brief.* (2017) 288:1–8. 29155689

[20] Djordjic V Radisavljevic S Milanovic I Bozic P Grbic M Jorga J WHO European Childhood obesity surveillance initiative in serbia: a prevalence of overweight and obesity among 6-9-year-old school children. *J Pediatr Endocrinol Metab.* (2016) 29:1025–30. 10.1515/jpem-2016-0138 27544722

[21] Yang S Guo B Ao L Yang C Zhang L Zhou J Obesity and activity patterns before and during COVID-19 lockdown among youths in China. *Clin Obes.* (2020) 10:12416. 10.1111/cob.12416 33009706 PMC7646045

[22] Pearce A Leonhardt C Vaidya C. Executive and reward-related function in pediatric obesity: a meta-analysis. *Child Obes.* (2018) 14:265–79. 10.1089/chi.2017.0351 29874102 PMC7141423

[23] Huang T Tarp J Domazet S Thorsen A Froberg K Andersen L Associations of adiposity and aerobic fitness with executive function and math performance in Danish adolescents. *J Pediatr.* (2015) 167:810–5. 10.1016/j.jpeds.2015.07.009 26256018

[24] Ross R Neeland IJ Yamashita S Shai I Seidell J Magni P Waist circumference as a vital sign in clinical practice: a consensus statement from the IAS and ICCR working group on visceral obesity. *Nat Rev Endocrinol.* (2020) 16:177–89. 10.1038/s41574-019-0310-7 32020062 PMC7027970

[25] Setiono F Guerra L Leung C Leak T. Sociodemographic characteristics are associated with prevalence of high-risk waist circumference and high-risk waist-to-height ratio in U.S. adolescents. *BMC Pediatr.* (2021) 21:215. 10.1186/s12887-021-02685-1 33941130 PMC8091763

[26] Wang Y Rimm E Stampfer M Willett W Hu F. Comparison of abdominal adiposity and overall obesity in predicting risk of type 2 diabetes among men. *Am J Clin Nutr.* (2005) 81:555–63. 10.1093/ajcn/81.3.555 15755822

[27] Liang J Matheson B Kaye W Boutelle K. Neurocognitive correlates of obesity and obesity-related behaviors in children and adolescents. *Int J Obes.* (2014) 38:494–506. 10.1038/ijo.2013.142 23913029 PMC4456183

[28] Schwartz D Leonard G Perron M Richer L Syme C Veillette S Visceral fat is associated with lower executive functioning in adolescents. *Int J Obes.* (2013) 37:1336–43. 10.1038/ijo.2013.104 23797144 PMC5061567

[29] Bugge A Möller S Westfall D Tarp J Gejl A Wedderkopp N Associations between waist circumference, metabolic risk and executive function in adolescents: a cross-sectional mediation analysis. *PLoS One.* (2018) 13:e0199281. 10.1371/journal.pone.0199281 29912925 PMC6005548

[30] Esteban-Cornejo I Tejero-González C Castro-Piñero J Conde-Caveda J Cabanas-Sanchez V Sallis J Independent and combined influence of neonatal and current body composition on academic performance in youth: the UP & DOWN Study. *Pediatr Obes.* (2015) 10:157–64. 10.1111/ijpo.239 24919886

[31] Veena S Hegde B Ramachandraiah S Krishnaveni G Fall C Srinivasan K. Relationship between adiposity and cognitive performance in 9-10-year-old children in South India. *Arch Dis Child.* (2014) 99:126–34. 10.1136/archdischild-2013-304478 24146284 PMC3982043

[32] Bi C. *Relationship between cardiorespiratory endurance and executive function and exercise intervention of tibetan children and adolescents in high altitude areas.* Shanghai: East China Normal University (2021).

[33] Department of Physical Education, Health and Art Education, Ministry of Education, National Student Physique and Health Research Group,. National student physique and health survey manual. *Beijing Higher Edu Pre.* (2014) 2014:17–24.

[34] Chen A Jiang R Ji X Tao B Zhu F Yan J. Effects of 8-week moderate fancy rope skipping training on executive function in preadolescent deaf children: a school-based experimental study. *J Sports Sci.* (2015) 36:105–9. 10.13598/j.issn1004-4590.2015.04.017

[35] Wylie S Ridderinkhof K Eckerle M Manning C. Inefficient response inhibition in individuals with mild cognitive impairment. *Neuropsychologia.* (2007) 45:1408–19. 10.1016/j.neuropsychologia.2006.11.003 17178419

[36] Smith E Jonides J. Working memory: a view from neuroimaging. *Cogn Psychol.* (1997) 3:5–42. 10.1006/cogp.1997.0658 9212720

[37] Salthouse T Atkinson T Berish D. Executive functioning as a potential mediator of age-related cognitive decline in normal adults. *J Exp Psychol Gen.* (2003) 132:566–94. 10.1037/0096-3445.132.4.566 14640849

[38] Liu Y Yin X Zhang F Li Y Bi C Sun Y Relationship between waist circumference and cardiorespiratory fitness in Chinese children and adolescents: results from a cross-sectional survey. *J Exerc Sci Fit.* (2022) 20:1–8. 10.1016/j.jesf 34868324 PMC8605195

[39] Egbert A Creber C Loren D Bohnert A. Executive function and dietary intake in youth: a systematic review of the literature. *Appetite.* (2019) 139:197–212. 10.1016/j.appet.2019.04.013 31014952

[40] Li L Zhang J Cao M Hu W Zhou T Huang T. The effects of chronic physical activity interventions on executive functions in children aged 3-7 years: a meta-analysis. *J Sci Med Sport.* (2020) 23:949–54. 10.1016/j.jsams.2020.03.007 32360243

[41] Zhan Z Ai J Ren F Li L Chu C Chang Y. Cardiorespiratory fitness, age, and multiple aspects of executive function among preadolescent children. *Front Psychol.* (2020) 11:1198. 10.3389/fpsyg.2020.01198 32587550 PMC7298134

[42] Textor J van der Zander B Gilthorpe M Liskiewicz M Ellison G. Robust causal inference using directed acyclic graphs: the R package ‘dagitty’. *Int J Epidemiol.* (2016) 45:1887–94. 10.1093/ije/dyw341 28089956

[43] Li Y Dai Q Jackson J Zhang J. Overweight is associated with decreased cognitive functioning among school-age children and adolescents. *Obesity.* (2008) 16:1809–15. 10.1038/oby.2008.296 18551126

[44] Davidson T Ramirez E Kwarteng E Djan K Faulkner L Parker M Retrieval-induced forgetting in children and adolescents with and without obesity. *Int J Obes.* (2022) 46:851–8. 10.1038/s41366-021-01036-5 35042933 PMC8967761

[45] Tomaso C James T Nelson J Espy K Nelson T. Longitudinal associations between executive control and body mass index across childhood. *Pediatr Obes.* (2022) 17:e12866. 10.1111/ijpo.12866 34725959 PMC8923908

[46] Mayer M Catalani F Fraire J Deltetto N Martín L Beneitez A Inhibitory control and obesity in adolescents: a prospective cohort study. *Appetite.* (2022) 171:105910. 10.1016/j.appet.2022.105910 35007663

[47] Khan N Baym C Monti J Raine L Drollette E Scudder M Central adiposity is negatively associated with hippocampal-dependent relational memory among overweight and obese children. *J Pediatr.* (2015) 166: 302–8.e1. 10.1016/j.jpeds.2014.10.008 25454939 PMC4308543

[48] Hassevoort K Khazoum S Walker J Barnett S Raine L Hammond B Macular carotenoids, aerobic fitness, and central adiposity are associated differentially with hippocampal-dependent relational memory in preadolescent children. *J Pediatr.* (2017) 183:108–14.e1. 10.1016/j.jpeds.2017.01.016 28189300

[49] Fan X Zhong Y Zhang L Li J Xie F Zhang Z. Abdominal obesity: an independent influencing factor of visuospatial and executive/language ability and the Serum Levels of Aβ40/Aβ42/Tau protein. *Dis Mark.* (2022) 2022:3622149. 10.1155/2022/3622149 35401883 PMC8993554

[50] Cheke L Bonnici H Clayton N Simons J. Obesity and insulin resistance are associated with reduced activity in core memory regions of the brain. *Neuropsychologia.* (2017) 96:137–49. 10.1016/j.neuropsychologia 28093279 PMC5317178

[51] Gonzales M Kaur S Eagan D Goudarzi K Pasha E Doan D Central adiposity and the functional magnetic resonance imaging response to cognitive challenge. *Int J Obes.* (2014) 38:1193–9. 10.1038/ijo.2014.5 24418893 PMC4097967

[52] Verdejo-García A Pérez-Expósito M Schmidt-Río-Valle J Fernández-Serrano M Cruz F Pérez-García M Selective alterations within executive functions in adolescents with excess weight. *Obesity.* (2010) 18:1572–8. 10.1038/oby.2009.475 20057376

[53] Delgado-Rico E Río-Valle J González-Jiménez E Campoy C Verdejo-García A. BMI predicts emotion-driven impulsivity and cognitive inflexibility in adolescents with excess weight. *Obesity.* (2012) 20:1604–10. 10.1038/oby.2012.47 22421897

[54] Smith E Hay P Campbell L Trollor JN. A review of the association between obesity and cognitive function across the lifespan: implications for novel approaches to prevention and treatment. *Obes Rev.* (2011) 12:740–55. 10.1111/j.1467-789X.2011 21991597

[55] Haapala E Lintu N Väistö J Robinson L Viitasalo A Lindi V Associations of physical performance and adiposity with cognition in children. *Med Sci Sports Exerc.* (2015) 47:2166–74. 10.1249/MSS.0000000000000652 26030086

[56] Holcke M Marcus C Gillberg C Fernell E. Paediatric obesity: a neurodevelopmental perspective. *Acta Paediatr.* (2008) 97:819–21. 10.1111/j.1651-2227.2008.00816.x 18430075

[57] Staiano A Abraham A Calvert S. Competitive versus cooperative exergame play for African American adolescents’ executive function skills: short-term effects in a long-term training intervention. *Dev Psychol.* (2012) 48:337–42. 10.1037/a0026938 22369339 PMC4097099

[58] Guo Y Yin X Sun Y Zhang T Li M Zhang F Research on environmental influencing factors of overweight and obesity in children and adolescents in China. *Nutrients.* (2021) 14:35. 10.3390/nu14010035 35010910 PMC8746339

